# Redistribution of joint moments is associated with changed plantar pressure in diabetic polyneuropathy

**DOI:** 10.1186/1471-2474-10-16

**Published:** 2009-02-03

**Authors:** Hans HCM Savelberg, Nicolaas C Schaper, Paul JB Willems, Ton LH de Lange, Kenneth Meijer

**Affiliations:** 1 Department of Human Movement Science, Faculty of Health, Medicine and Life Sciences, Maastricht University, Maastricht, the Netherlands; 2 Nutrition and Toxicology Research Institute, Faculty of Health, Medicine and Life Sciences, Maastricht University, Maastricht, the Netherlands; 3 Department of Internal Medicine, University Hospital Maastricht, Maastricht, the Netherlands; 4 Centre of Expertise Health and Mobility, Fontys University of Applied Sciences, Eindhoven, the Netherlands

## Abstract

**Background:**

Patients with diabetic polyneuropathy (DPN) are often confronted with ulceration of foot soles. Increased plantar pressure under the forefoot has been identified as a major risk factor for ulceration. This study sets out to test the hypothesis that changes in gait characteristics induced by DPN related muscle weakness are the origin of the elevated plantar pressures.

**Methods:**

Three groups of subjects participated: people diagnosed with diabetes without polyneuropathy (DC), people diagnosed with diabetic polyneuropathy (DPN) and healthy, age-matched controls (HC). In all subjects isometric strength of plantar and dorsal flexors was assessed. Moreover, joint moments at ankle, knee and hip joints were determined while walking barefoot at a velocity of 1.4 m/s. Simultaneously plantar pressure patterns were measured.

**Results:**

Compared to HC-subjects, DPN-participants walked with a significantly increased internal plantar flexor moment at the first half of the stance phase. Also in DPN-subjects the maximal braking and propelling force applied to the floor was decreased. Moreover, in DPN-subjects the ratio of forefoot-to-rear foot plantar pressures was increased. Body-mass normalized strength of dorsal flexors showed a trend to be reduced in people with diabetes, both DC and DPN, compared to HC-subjects. Plantar flexors tended to be less weak in DC compared to HC and in DPN relative to DC.

**Conclusion:**

The results of this study suggest that adverse plantar pressure patterns are associated with redistribution of joint moments, and a consequent reduced capacity to control forward velocity at heel strike.

## Background

Diabetic foot ulceration is one of the major complications of diabetes, leading to high morbidity, poor quality of life and high costs [1]. The pathogenesis of these ulcers is usually multifactorial, but in many patients diabetic polyneuropathy plays a pivotal role [2]. Several studies have shown that increased plantar pressures play an important role in the development of plantar forefoot ulcers in diabetic patients with neuropathy [3,4]. Several different mechanisms can contribute to this increase in plantar forefoot pressure. Structural changes in an insensitive foot, such as prominent metatarsal heads, toe deformities or Charcot deformity can be an important cause of elevated pressures. Moreover, joint abnormalities such as limited mobility at the ankle are associated with increased pressures.

Muscle weakness [5-10] and atrophy [9,11,12] are characteristics of the sensory-motor loss of diabetic neuropathy. As peripheral polyneuropathy proceeds from distal to proximal, it is expected that muscle function is affected in a distoproximal order. As a consequence, an imbalance between muscles can be expected that will influence intermuscular coordination during standing and walking. Indeed, patients with diabetic neuropathy have abnormal gait performance which is characterized by reduced gait velocity, longer stance (contact) phase duration, smaller range of motions in joints, delayed muscle activation patterns and reduced joint moments [13-19].

In normal walking, the centre of pressure (CoP) under the foot transfers gradually from the heel to the forefoot. Several authors found that in diabetic patients with neuropathy the pressures under the forefoot are relatively increased compared to those under the heel [20,21]. However, the mechanism of this relative increase of forefoot pressure is unclear. Abboud et al. [22] investigated the activation patterns of lower leg muscles and plantar pressures in diabetic patients and suggested that increased plantar pressures result from lower limb muscle dysfunction. These authors found an earlier loading of the forefoot and at the same time reduced ankle dorsal flexor activation in diabetic patients. Similar changes were reported by Sacco and Amadio [18]. It remains unclear, however, how abnormal lower limb muscle activation results in increased plantar pressures. A possible explanation can be deduced from the work by Van Ingen Schenau et al. [23,24], who reported that the magnitude and direction of the force applied to the ground and thereby the transfer of the CoP under the foot is determined by the coordinated action of multiple muscles. Based on this and the above mentioned consideration on muscle weakness, we hypothesize that the increase in plantar pressures in neuropathy is the consequence of muscle weakness, which will lead to impaired intermuscular coordination, which in its turn affects the transfer of the CoP from heel to forefoot. As far as we know, the interrelationship between muscle function, gait performance and plantar pressure patterns has not yet been investigated in patients with diabetic polyneuropathy. The effects of diabetes and diabetic polyneuropathy on muscle function [5,8,12], gait performance [14,15,18,22,25] and plantar pressures [20,21,26] were considered separately in earlier studies. It is the aim of this study to identify the cascade of relationships that link muscle weakness to plantar pressure. Evidence for such a cascade would provide crucial leads for future interventions to improve muscle strength in order to reduce elevated plantar pressures and related disabilities.

## Methods

### Subjects

Eight diabetic patients with polyneuropathy (DPN), ten diabetic controls without neuropathy (DC) and ten healthy, age-matched controls (HC) participated (Table 1). Clinical diabetic polyneuropathy was diagnosed based on a standardized clinical examination which included sensory testing, tendon reflexes and muscle strength in the lower extremities [27]. Patients were recruited from the outpatient clinic of the university hospital Maastricht and healthy controls from the community. Subjects with foot deformities or active ulceration were excluded; additional exclusion criteria were: limited mobility due to severe joint problems, angina pectoris or cardiac failure NYHA Class 2 or higher, myocardial infarction within 1 year, BMI>35 and orthopaedic or neuromuscular disease other than diabetic polyneuropathy. The study was approved by the ethical committee of the university hospital Maastricht; all subjects gave written informed consent.

**Table 1:** Subject characteristics

	**HC**	**DC**	**DPN**	p-value
**Sex (female:male)**	2:8	3:7	1:7	NA

**Age (years)**	72.4(6.0)	60.5(6.9)	68.9(6.3)	**0.003**

**Body mass (kg)**	71.5(10.0)	81.3(13.3)	84.1(10.9)	0.062

**Body length (m)**	1.70(0.07)	1.67(0.10)	1.73(0.06)	0.332

**Leg length (m)**	0.78(0.06)	0.77(0.08)	0.82(0.04)	0.311

**BMI (kg/m^2^)**	24.7(2.9)	29.2(3.7)	28.0(3.2)	**0.008**

**Vibration Perception Threshold (V)**	21.8(8.4)	17.9(6.7)	38.1(8.4)	**0.002**

**HbA1c (%)^1^**		8.8(2.0)	8.1(0.6)	0.360

**Duration of diabetes (years)^1^**		10.2(5.4)	19.0(13.6)	0.314

Values represent means(standard deviations)

^1 ^DC: n = 5; DPN n = 6

### Experimental procedure

Subjects visited the laboratory twice, for a dynamometer test and for a gait test. To estimate whether subjects could safely participate in the tests, blood glucose concentration was measured before and after each measurement.

To determine strength of plantar and dorsal flexors of the right leg, subjects performed in a dynamometer (Cybex II, Ronkonkoma, N.Y., USA) maximal, isometric, voluntary contractions at 25 combinations of knee and ankle joint angles. Each contraction lasted about 3 seconds. To prevent fatiguing, three minutes of rest were allowed between subsequent contractions. Contractions were performed at five knee and five ankle joint angles that covered the whole range of joint motion of each individual. At each combination of knee and ankle joint angles one contraction was performed. The range of joint motion at knee and ankle did not differ between subject groups.

As a measure of plantar sensitivity the vibration perception threshold >25 V was determined on the hallux [28].

For gait analysis markers were attached to the skin over the head of the fifth metatarsal bone, the heel of the foot, the lateral malleolus, the epicondyle of the femur and the greater trochanter of the right leg. A 2D, 50 Hz video system (Adimec, Eindhoven, The Netherlands) registered the marker positions in the sagittal plane. The spatial resolution of the video system was 3.85 mm/pixel; spatial accuracy of marker position is approximately 25% of this: 1 mm. A pair of electromyography (EMG) electrodes (K_lab, Amsterdam, The Netherlands) was placed halfway on the line connecting the greater trochanter and the sacrum, over the belly of gluteus maximus to record the activation of the underlying muscle tissue. Subjects walked barefoot across a 12-meter walkway with halfway an embedded force plate (Kistler type 9281A: accuracy of vertical force component: 2%; accuracy of horizontal force component: 4%; accuracy of point of application of force vector: 8 mm, Kistler Instrumente AG, Wintherthur, Switzerland) and a pressure platform (spatial resolution: two sensors/cm^2^; Novel GmbH, Munich, Germany). The pressure platform, which was flush with the walkway surface, was placed on top of the force platform. Since gait velocity influences plantar pressures [29,30] and gait performance [31], subjects were asked to walk five times at the same velocity of 1.4 m/s. The force platform was sampled at 1000 Hz, the pressure platform at 70 Hz. To obtain an estimate of the effort that this gait velocity required, subjects were also asked to walk five times at a self-selected velocity. The self-selected velocity conditions preceded the trials at the imposed velocity.

Subjects practiced on the walkway to make sure they would step on the platforms with their right foot without altering their walking pattern. If yet, the foot was not placed properly or if a subject made visually obvious alterations to hit the force platform, the particular trial was discarded. The measurements were continued until five trials with a correct foot placement and gait velocity were collected.

### Data-analysis

A second degree polynomial was fitted through the joint moments at the different joint angles (cf. [32]) and the maximal joint moment was defined as the maximal value of this polynomial. To evaluate to what extend muscle strength changes reflect the effects of diabetic polyneuropathy only independent of increased effort during activities of daily living that result from changes in body mass, muscle strength measurements were also normalized for body mass.

Gait velocity was calculated as the average horizontal velocity of the greater trochanter marker. Stride time was determined as the time between the onsets of two consecutive EMG-bursts of the gluteus maximus.

The leg was modeled as three rigid segments (foot, lower leg and thigh). Position data of the markers were used to determine the position of segments, and subsequently -by differentiation- (angular) velocity and acceleration.

**Figure 1:**
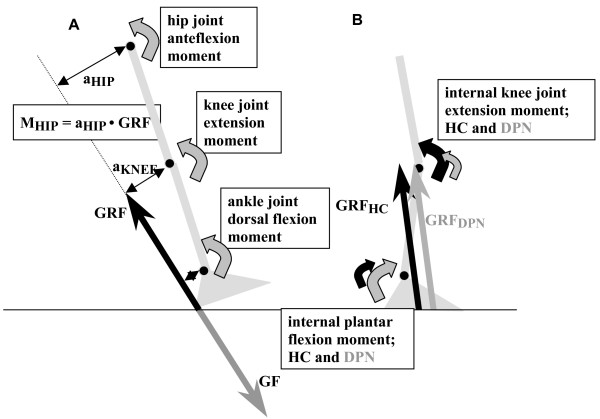
**a: A theoretical scheme of the foot, the lower leg and thigh, and the force and joint moments that are present during heel strike**. During the stance phase the foot exerts a force on the ground (GF, grey, downward arrow). The reaction force to this GF is the Ground Reaction Force (GRF, black upward arrow). The GRF is the force that brakes and propels the human body and that exerts extending and flexing moments on the respective joints. In the first half of the stance phase the GRF brakes the forward velocity, in the second have it propels the body. By measuring this GRF and its joint moment arms the external joint moments can be calculated, as illustrated: M_HIP _= a_HIP _•GRF. Like the GRF is the opposite of the force applied to the floor (GF), the external joint moments are the opposite of the moments generated internally around the joints by the muscles (Figure 4a-c). In the figure the curved arrows represent these internal moments. During normal walking velocities, the GRF is the major determinant of the external joint moments; and oppositely muscle function is a major determinant of GRF. **1b: **This figure shows the leg and the GRF at about 40% of the stance phase; the GRF is directed in front of the ankle joint and behind the knee joint. Consequently the GRF has a dorsal flexion moment at the ankle joint and a flexion moment at the knee and the internal moments will be plantar flexion and knee joint extension. A forward displacement of the GRF will result in a smaller moment arm at the knee joint and a larger moment arm at the ankle joint, this will result in increase plantar flexion (Figure 4a) and decreased knee joint moments (Figure 4b). The black arrow represents the GRF in HC (GRF_HC_), the grey one the GRF in DPN (GRF_DPN_),. The curve arrows represent the internal joint moments at the knee and ankle joint (HC: black curved arrows, DPN: grey curved arrows).

During walking, movement is caused by moments of force generated by muscles around ankle, knee and hip joint (Figure 1a). Based on the ground reaction force (GRF), the calculated accelerations of the body segments (foot, lower and upper leg) and the estimated inertial parameters of these segments, an inverse dynamics approach was applied to calculate net internal joint moments. Based on body mass and segment length inertial parameters of segments were estimated [33]. Segment length was determined by the distance between relevant markers. Net joint moments represent the summed effect of all structures that produce a moment across a joint. Summation of the moments at ankle, knee and hip joint resulted in the support moment [34]. The support moment is considered to represent the overall moment generated in the limb and gives quantitative information about the supporting and propelling muscle effort [35].

Only when the GRF was >300 N, the point of application of the GRF could be measured validly. Consequently, inverse dynamics could not be performed for the entire stance phase. In the graphical data presented, this results in graphs ranging typically between 10 and 90% of the stance phase.

As we were especially interested in changes in gait pattern due to diabetic polyneuropathy and not due to differences in body mass, GRF data (for-aft and vertical components) and joint moment curves were normalized for stance phase duration and body mass; average curves were calculated for each participant. For each curve the minimal and maximal values were determined. In addition, for the fore-aft component of GRF the braking and propulsive impulses were assessed. For the vertical component of GRF and the support moment curves, which both have a typical M-shape, the maximal values at both peaks and the minimal value between the peaks were determined. The ankle joint moment reached a plateau between approximately 30 and 50% of the stance phase; therefore, the value at 40% of the stance phase was also determined to evaluate the level of this plateau. Finally, the area under the ankle joint moment curve was calculated, which represents the joint impulse. This impulse is a measure for the amount of work generated around the ankle joint.

Using a PRC-mask, peak plantar pressures and the plantar pressure time integrals were calculated for ten anatomical areas of the foot [36]. Pressure ratios were calculated for different combinations of forefoot (heads of the first metatarsal, the second metatarsal and of the area of the third until the fifth metatarsal) to heel (medial and lateral) areas. To monitor the forward transfer of the Centre of Pressure (CoP), the time (percentage of stance phase) at which the CoP entered one of the midfoot, metatarsal and toe areas was determined.

### Statistical analysis

The non-parametric Kruskal-Wallis test was applied to investigate whether muscle strength and gait performance variables differed between the groups. P = 0.05 was chosen as level of significance. If this test revealed a significant effect of the groups on a variable, Mann-Whitney U-tests were applied as a post hoc test. For the latter test the Bonferroni-correction was adapted to correct the level of significance; p-values smaller than 0.017 were considered significant.

## Results

### Strength of lower leg muscle groups

**Figure 2:**
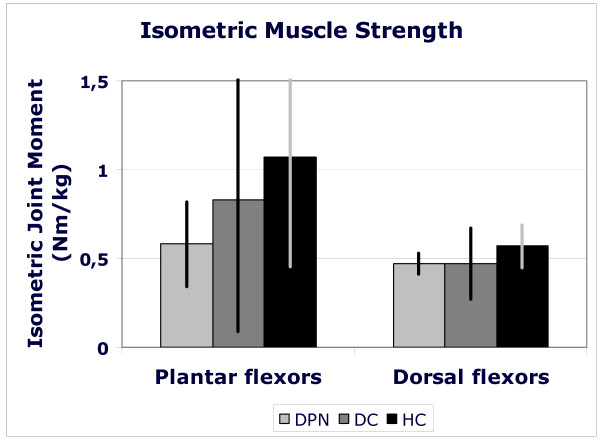
**Maximal, voluntary, isometric strength of plantar and dorsal flexor muscle groups for health elderly (black bars), people with diabetes without polyneuropathy (dark grey bars) and people with diabetic polyneuropathy (light grey bars)**. Muscle strength is expressed as the joint moment exerted in a dynamometer normalized for body mass (Nm/kg). Bars represent mean values for a group; standard deviations are presented as vertical lines.

Body-mass-normalized dorsal flexor moment tended to be lower in both diabetic groups compared to healthy controls (p = 0.128). The average normalized dorsal flexor moments were 0.57, 0.47 and 0.47 Nm/kg (HC, DC and DPN). For normalized plantar flexion strength a non-significant trend (p = 0.153) was observed: 1.07, 0.83 and 0.58 Nm/kg in the HC, DC and DPN patients, respectively (Figure 2). Absolute strength of plantar flexors (HC: 78.2 ± 46.6 Nm; DC: 66.7 ± 53.5 Nm; DPN: 50.3 ± 28.6 Nm) and of dorsal flexors did not differ between groups (HC: 40.8 ± 11.0 Nm; DC: 38.1 ± 15.4 Nm; DPN: 39.5 ± 8.2 Nm).

### Plantar sensitivity

DPN-participants had a significantly higher vibration perception threshold than the other subjects (p = 0.002; Table 1).

### Gait characteristics

Imposed gait velocities did not differ significantly between groups (HC: 1.44 ± 0.13; DC: 1.35 ± 0.10; DPN: 1.40 ± 0.12 m/s). In addition, no differences were observed in cadence or stride length (data not shown). The self-selected velocity was for all subjects lower than the imposed velocity. The ratio of the imposed velocity over the self-selected velocity was larger in diabetic polyneuropathy participants than in healthy and diabetic controls (HC: 1.22 ± 0.20; DC: 1.30 ± 0.13; DPN: 1.40 ± 0.09; DC vs DPN: 0.050; HC vs DPN: p = 0.051), indicating that the DPN participants had to walk relatively fast in the test condition.

### Ground reaction force patterns

**Figure 3:**
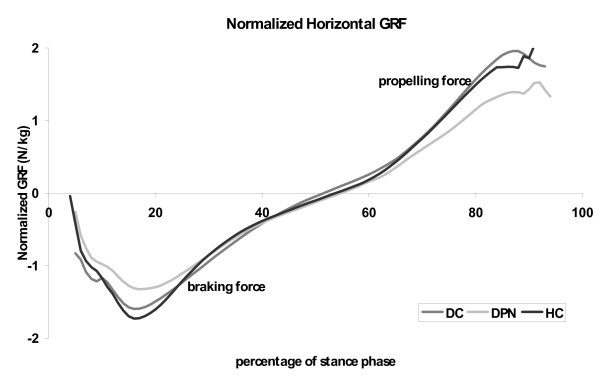
**Fore-aft component of the ground reaction force for health elderly (black lines), people with diabetes without polyneuropathy (dark grey lines) and people with diabetic polyneuropathy (light gray lines)**. Graphs represent averages values for each group.

The body-mass-normalized braking force differed between groups (p = 0.041); Figure 3), with a lower braking force in DPN than in diabetic and healthy subjects. Also the maximal propelling force was significantly smaller in DPN than in both other groups (p-0.012; Figure 3). The vertical component of the GRF did not differ between the groups.

### Joint moments during walking

**Figure 4:**
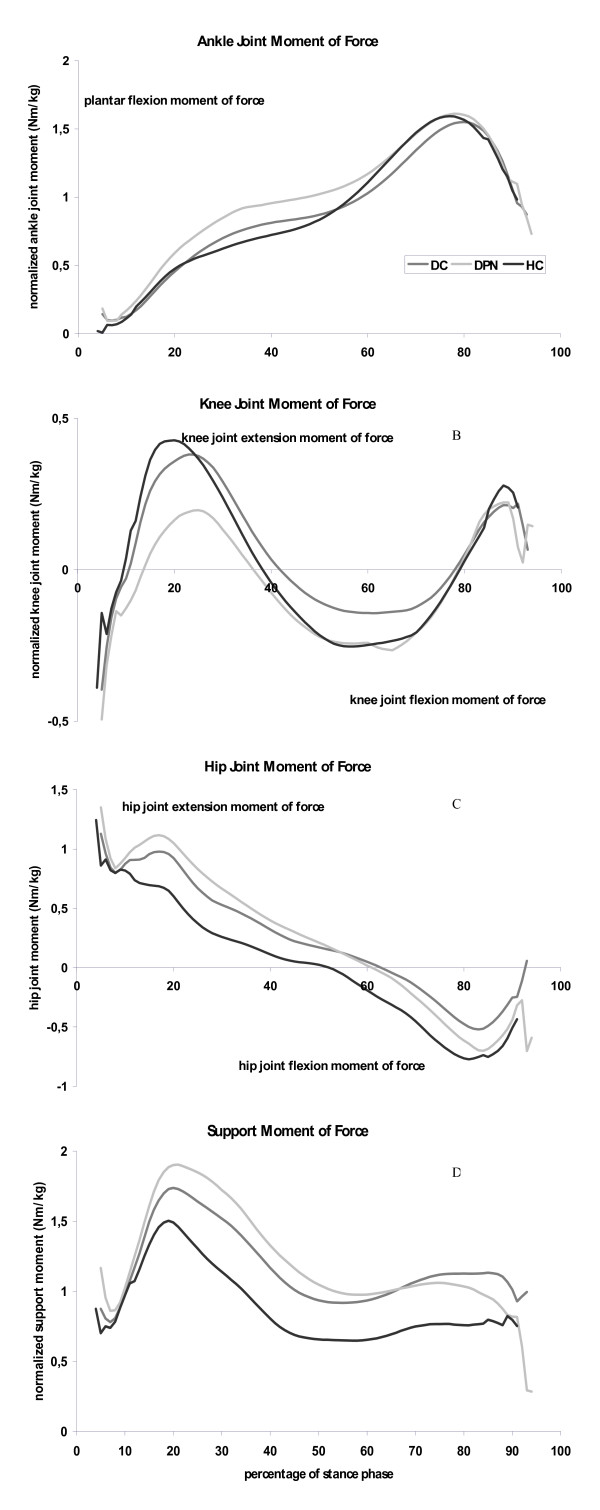
**Joint moment patterns as a function of the stance phase for health elderly (black lines), people with diabetes without polyneuropathy (dark grey lines) and people with diabetic polyneuropathy (light gray lines)**. Graphs represent the internal joint moments, and are averaged values for each group. **4a: **Ankle joint moment, positive values indicate plantar flexor muscle moments. **4b: **Knee joint moment, positive values indicate knee joint extensor muscle moments. **4c: **Hip joint moment, positive values indicate hip joint extensor muscle moments. **4d: **Support moment, representing the summation of ankle, knee and hip joint moments.

In DPN-subjects the plantar flexion moment during the first half of the stance phase: at 40% of the stance phase was approximately 30% higher than in HC subjects (Table 2; p = 0.027; DPN vs HC: p = 0.013). As a consequent of this, the plantar flexion impulse at the ankle joint trended to be higher in DPN than in both other groups (Figure 4a; Table 2; p = 0.155). Maximal plantar flexion moments did not differ significantly between the groups.

**Table 2:** Parameters of muscle strength and of dynamic variables characterizing gait pattern of the subjects

	**HC**	**DC**	**DPN**	p-value
***Isometric muscle strength***				

**Body-mass-normalized plantar flexion (Nm/kg)**	1.07(0.62)	0.83(0.74)	0.58(0.24)	0.153
**Body-mass-normalized dorsal flexion (Nm/kg)**	0.57(0.12)	0.47(0.20)	0.47(0.06)	0.128

***Ground reaction force***				

**Normalized maximal braking force (N/kg)**	-1.74(0.45)	-1.69(0.29)	-1.30(0.31)	**0.041**
**Normalized, maximal propelling force (N/kg)**	1.78(0.50)	2.02(0.34)	1.42(0.35)	**0.012**
**Normalized braking impulse (Ns/kg)**	-0.24(0.08)	-0.26(0.06)	-.24(0.5)	0.683
**Normalized propelling impulse (Ns/kg)**	0.20(0.06)	0.26(0.06)	0.19(0.09)	0.085

***Joint Moment characteristics***				

**Normalized Maximal Plantar flexion Moment (Nm/kg)**	1.59(0.17)	1.51(0.21)	1.64(0.26)	0.647
**Normalized plantar flexion moment at 40% of stance phase (Nm/kg)**	0.70(0.12)	0.82(0.17)	0.97(0.21)	**0.027**
**Normalized plantar flexion impulse (Nms/kg)**	0.53(0.07)	0.55(0.10)	0.70(0.20)	0.155
**Normalized maximal knee joint extension moment (Nm/kg)**	0.45(0.36)	0.42(0.22)	0.23(0.30)	0.249
**Normalized maximal knee joint flexion moment (Nm/kg)**	-0.28(0.27)	-0.16(0.11)	-0.29(0.21)	0.505
**Normalized maximal hip joint extension moment (Nm/kg)**	0.85(0.40)	1.09(0.25)	1.08(0.39)	0.230
**Normalized maximal hip joint flexion moment (Nm/kg)**	-0.70(0.12)	-0.55(0.26)	-0.75(0.36)	0.233

***Support moment curve***				

**Normalized, First Maximal support Moment (Nm/kg)**	1.52(0.47)	1.84(0.33)	1.87(0.32)	0.150
**Normalized midstance minimal support moment (Nm/kg)**	0.60(0.21)	0.85(0.31)	0.93(0.26)	0.056
**Normalized, second Maximal supportMoment (Nm/kg)**	0.86(0.20)	1.20(0.44)	1.18(0.33)	0.065
**Normalized 'impulse' of support moment (Nms/kg)**	0.54(0.17)	0.75(0.22)	0.87(0.22)	**0.021**

Values represent means(standard deviations)

The characteristics of the knee and hip joint moment curves did not differ between the groups. The impulse of the total support moment and the minimum between both peaks were larger in both diabetic patients compared to HC subjects (Figure 4d; Table 2; p = 0.021 (impulse) and p = 0.056 (local minimal value)).

### Plantar pressure patterns

**Figure 5:**
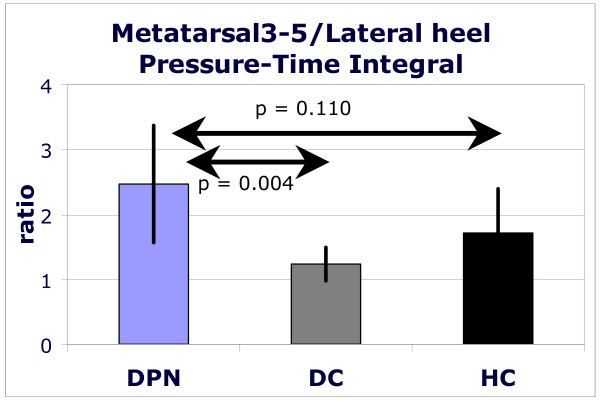
**The ratio of the time-pressure integral for the area under the lateral metacarpal heads (3–5) over area under the lateral part of the heel for healthy elderly (black bars), people with diabetes without polyneuropathy (dark grey bars) and people with diabetic polyneuropathy (light grey bars)**. Ratio larger than 1 indicate time-pressure integrals under the fore foot to be larger than those under the heel. Bars represent mean values for a group; standard deviations are presented as vertical lines.

**Figure 6:**
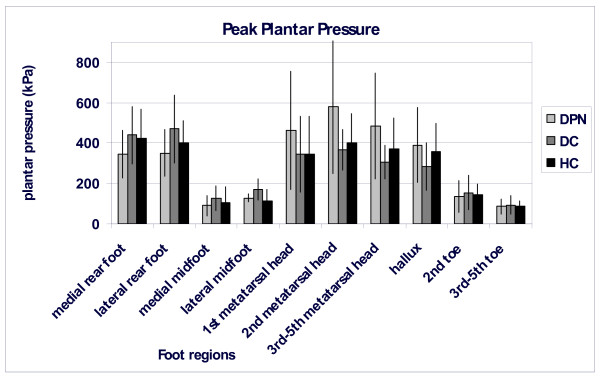
**Peak pressure patterns (kPa) for each of the ten regions for all three groups (black: healthy controls; dark grey: diabetic controls; light grey: people with diabetic polyneuropathy)**. Bars represent mean values for a group; standard deviations are presented as vertical lines.

In DPN compared to HC and DC, the ratios of peak plantar pressures and of time-integrated pressures were significantly increased for the region of the heads of the three lateral metatarsals over the lateral rear foot (peak pressure: p = 0.010; pressure time integral p = 0.013; figure 5). Other ratios did not differ significantly. In the DPN subjects the CoP entered the forefoot area earlier than in both other groups, also the CoP stayed longer in this area, but these temporal differences were not statistically significant (data not shown). The forward shift of the pressure patterns in people with diabetic polyneuropathy is also illustrated in Figure 6. This figure contains peak pressure pattern for each of the ten foot regions and for each group.

## Discussion

This study sought to identify associations between reduced muscle strength, adaptations in gait dynamics and changes in plantar pressure patterns in relation to diabetes and diabetic polyneuropathy. The innovative aspect of this study is the combination of measurements at three levels; we assessed in all subjects muscle strength, joint moments during normal walking and plantar pressure patterns. A second aspect in which this study differed from previous reports is the standardized velocity. The results of this study suggest that due to a redistribution of joint moments a faster forward transfer of the centre of pressure underneath the foot during the stance phase occurred. As a consequence the forefoot will be loaded earlier and longer. The study was not conclusive with respect to the question whether redistribution of the joint moments resulted from reduced muscle strength; also reduced sensitivity of the foot sole might play a role in this.

With respect to gait dynamics, a redistribution of joint moments was found in DPN participants. The plantar flexion moment in the first half of the stance phase was significantly higher in DPN. Also during the first half of the stance phase, the knee joint extension moment tended to be lower in DPN participants. In a study that did not control gait velocity, Kwon et al. [15] found similar results comparing subjects with and without DPN, but in addition they reported decreased maximal plantar flexion moments and knee joint extension moments. Also Mueller et al. [17] found reduced maximal plantar flexion moments in diabetic polyneuropathy in an experimental setting uncontrolled for walking velocity. These authors suggested that this reduction was caused by a decline in plantar flexor strength; however it could also have resulted from the lower gait velocity that people with diabetic polyneuropathy tend to choose. As in our study the maximal plantar flexor moment is not affected, limitations in gait performance do not seem to arise from this muscle group. The redistribution of joint moments in diabetic polyneuropathy differs from that reported for elderly relative to young adults. In comparison to younger adults, elderly experience a redistribution from plantar flexors moments at the ankle joint to higher hip joint extension moments [35,37]. These differences in joint moment redistribution suggest that in diabetic polyneuropathy and ageing specific patterns of muscle wasting develop that require specific adaptations in gait dynamics.

The higher forefoot-to-rear foot plantar pressure ratio in DPN is in agreement with previous studies [20,21,26] that did not control gait velocity.

Diabetic controls and participants with diabetic polyneuropathy had lower leg muscles that tended to be weak with respect to their body mass. Absolute lower leg muscle strength did not differ between HC and diabetic subjects with and without polyneuropathy. This is in contrast to previous studies that reported a 15–40% decrease of absolute strength for these muscles [5-7,9,10].

In conclusion, people with diabetic polyneuropathy seem to put more effort in walking at a given velocity: they tend to have reduced relative muscle strength, their self-selected gait velocity is relatively low and they generate higher total extending joint moments (support moment) than DC and HC participants. Simultaneously, a relative forward shift of the pressure under the foot was found. The question is now, is a redistribution of joint moments the cause of increased plantar pressures? It has been suggested that limited mobility at the ankle joint, claw/hammer toe deformity and increased maximal plantar flexor moments are causes of increased plantar pressure [38-40], but all these factors did not differ between our groups.

Interestingly, the changes in plantar flexion moment occurred during the first half of the stance phase. At a normal walking speed, the GRF is the major determinant of the external ankle joint moments (figure 1A). As illustrated in figure 1B a higher plantar flexion moment will result in a faster forward translation of the GRF. At the same time a lower knee joint extension moment would have been expected too in DPN. In this study we reported a non-significant reduction of the knee joint extension moment in DPN.

The stance phase of gait is characterized by subsequent braking and propulsive parts. Consequently, velocity is reduced in the first half of the stance phase in normal gait and increases again during the second half. As mentioned, the changes in joint moments occurred especially in the first half of the stance phase, suggesting that the changes in joint moments represent a limited ability to brake in the first half of the stance phase. This was confirmed by the significantly lower braking (and propelling) peaks of the horizontal component of the ground reaction force (Figure 3). Meier et al [41] reported similar results for elderly patients with diabetic polyneuropathy. When the deceleration of the forward velocity is reduced, the centre of mass and the centre of pressure will remain traveling forwardly faster. Due to a faster forward transfer of the GRF the heel area will be unloaded earlier in the stance phase and the forefoot will be confronted with an earlier and longer lasting loading. This would result in an increased forefoot-to-rear foot ratio, as also observed by other authors [20,21]. Although we did find an increased forefoot-to-rear foot ratio, our data were not accurate enough to establish a faster forward shift of the centre of pressure (CoP) under the foot. We estimate that both the limited number of subjects and the way we assessed the CoP-velocity negatively affected the discriminative power for this variable.

The other side of the cascade of relationships that we wanted to investigate relates to the question whether the redistribution of joint moments has been caused by muscle weakness. In this study only strength of lower leg muscles was measured; a non-significant decrease of plantar and dorsal flexor strength was found. Based on this it cannot be concluded that muscle weakness, operationalized as maximal strength, caused the adaptations in gait dynamics. Other factors that contribute to muscle function, like rate of force development and level of activation, might have contributed too. Changes in the activation of muscle might arrange from affected nerve function, *i.e*. conductive velocity, or decreased proprioceptive or sensory information. In DPN-participants sensitivity was less compared to both other groups, as a result of this it can be assumed that proprioception in DPN will be disturbed and that consequently proper activation of muscles might be hampered. Also adaptations in more proximal muscles cannot be excluded as the origin of the adapted gait. In previous publications it has been suggested that disturbed gait performance is associated with delayed activity of dorsal flexors like tibialis anterior [22] and knee joint extensors, *i.c*. vastus medialis [18].

With respect to the accuracy of the technical equipment the determination of the point of application of the GRF seems to be most critical. Its accuracy (8 mm) was considerably smaller than relevant differences in joint moment arms (2–3 cm) that were found between the groups. So this accuracy can be considered to be sufficient. To assess plantar pressure pattern a pressure plate with a resolution of two sensors/cm^2 ^was used. If the sensor area is larger than the area of relevant pressure peaks, the real pressure will be underestimated. As shown among others by Cavanagh and Ulbrecht [42] such a two-sensors/cm2 system is reliable to assess plantar pressure in diabetic gait. The number of subjects in this study was small. Due to this the statistical power was low. Another limitation was the age difference between HC and DPN participants and the DC group. Although the effect of polyneuropathy on muscle weakness is known to be higher than that of ageing, a better match would have contributed to a higher statistical power. Also subjects were not matched for body mass; this was the consequence of matching participants for leg length. In spite of these methodological limitations, the present study contributed to a new hypothesis stating that due to a redistribution of joint moments braking of the forward transfer of the centre of pressure is hampered, as a consequence the centre of pressure underneath the foot might travel faster to the forefoot, resulting in an earlier loading of the forefoot. The two alternative hypotheses that have been suggested for the key factor underlying this joint moment redistribution in DPN are weakness of proximal muscle like knee extensors or reduced plantar sensitivity. With more and better matched subjects the statistical power might have been higher, and the hypothesis might have been more strongly underpinned. However, to test whether the proposed cascade of relationships really exists and whether the various findings of this study are not just an association of phenomena with a common underlying cause an intervention study will be necessary. The importance of the present study is that it provides evidence for such a cascade and thus helps to direct future intervention studies.

## Conclusion

In conclusion, people with diabetic polyneuropathy had a higher forefoot-to-rear foot ratio of plantar pressures than diabetic and healthy controls. It was suggested that adapted plantar pressure patterns are associated to a redistribution of joint moments around the ankle joint during walking. A hypothesis has been presented relating muscle weakness and adapted joint moments to changes in plantar pressure patterns. In addition, a trend to relatively reduced lower leg muscle strength in people with diabetes was found. This might be one of the factors underlying adaptations in gait dynamics.

## Competing interests

The authors declare that they have no competing interests.

## Authors' contributions

HHCMS contributed to the conception and design of this study, also he took care of data analysis and initial interpretation of data and wrote the first draft of the manuscript. NCS, TLHdL and KM were involved in the conception of the study and in the critical revision of initial versions of this manuscript. PJBW lead the data-acquisition and set up analysis and data processing. All authors read and approved the final version of the manuscript.

## Pre-publication history

The pre-publication history for this paper can be accessed here:


